# Identification of PCBP1 as a Novel Modulator of Mammalian Circadian Clock

**DOI:** 10.3389/fgene.2021.656571

**Published:** 2021-03-26

**Authors:** Yaling Wu, Haijiao Zhao, Eric Erquan Zhang, Na Liu

**Affiliations:** ^1^Hubei Engineering Research Center of Special Wild Vegetables Breeding and Comprehensive Utilization Technology, Hubei Normal University, Huangshi, China; ^2^Hubei Key Laboratory of Edible Wild Plants Conservation and Utilization, Hubei Normal University, Huangshi, China; ^3^National Demonstration Center for Experimental Biology Education, Hubei Normal University, Huangshi, China; ^4^College of Life Sciences, Hubei Normal University, Huangshi, China; ^5^National Institute of Biological Sciences, Beijing, China

**Keywords:** circadian clock, period shortening, clock modifier, PCBP1, mechanism

## Abstract

The circadian clock governs our daily cycle of behavior and physiology. Previous studies have identified a handful of core clock components and hundreds of circadian modifiers. Here, we report the discovery that poly(C)-binding protein 1 (PCBP1), displaying a circadian expression pattern, was a novel circadian clock regulator. We found that knocking down *PCBP1* resulted in period shortening in human U2OS cells, and that manipulations of *PCBP1* expression altered the activity of CLOCK/BMAL1 in an E-box-based reporter assay. Further mechanistic study demonstrated that this clock function of PCBP1 appears to work by enhancing the association of Cryptochrome 1 (CRY1) with the CLOCK/BMAL1 complex, thereby negatively regulating the latter’s activation. Co-immunoprecipitation of PCBP1 and core clock molecules confirmed the interactions between PCBP1 and CRY1, and a time-course qPCR assay revealed the rhythmic expression of *PCBP1* in mouse hearts *in vivo*. Given that the RNA interference of *mushroom-body expressed* (*mub*), the poly(rC) binding protein (PCBP) homolog of *Drosophila*, in the clock neurons also led to a circadian phenotype in the locomotor assay, our study deemed PCBP1 a novel clock modifier whose circadian regulatory mechanism is conserved during evolution.

## Introduction

Poly(rC) binding proteins (PCBPs) are generally known as RNA-binding proteins. These proteins comprise two subsets: PCBPs (1–4; also referred to as α-CPs or hnRNP E 1–4) and hnRNPs K/J in mammalian cells, the homolog of which is *mushroom-body expressed* (*mub*) in *Drosophila*. The PCBP family is related evolutionarily by the common feature of poly(C)-binding specificity and three highly conserved hnRNP K homology (KH) domains (Makeyev and Liebhaber, 2002). PCBPs perform multiple biological processes through the conserved characteristics, including mRNA stabilization, transcriptional regulation, and translational silencing (Huo and Zhong, 2008; Zhang et al., 2020a). At present, poly(C)-binding protein 1 (PCBP1), which exhibits 82% similarity at amino acid level to poly(C)-binding protein 2 (PCBP2) (Guo and Jia, 2018), has been studied in the greatest detail. The ability of PCBP1 to recognize and bind poly(C) DNA and RNA sequences *via* KH domains is critical for their function in mammalian cells. As such, PCBP1 binds to various proteins, including iron-containing polyamine pathway dioxygenase ADI1 and signal transducer and activator of transcription 3 (STAT3), and then participates in iron homeostasis, tumorigenesis, and metastasis (Yanatori et al., 2020; Zhang et al., 2020b). Together, these studies describe the complexity of distinct interacting factors that are mediated by PCBP1, which revealed that PCBP1 participate in multiple biological processes.

In the dynamic homeostasis of living organisms, the circadian clock drives endogenous rhythms of multiple physiological processes (Zhang and Kay, 2010). In addition, disruption of the circadian rhythm plays a key role in numerous chronic diseases, such as cancer and metabolic syndrome (Roenneberg and Merrow, 2016; Jagannath et al., 2017). The core circadian oscillator in mammals is composed of a transcription-translation feedback loop (TTFL) with CLOCK and BMAL1 as positive factors and PER and CRY as negative regulators. An additional feedback loop exits with the ROR transcriptional activators and the dimeric REV–ERB repressors regulating the Bmal1 gene, the expression of which, in turn, controls the transcription of ROR and REV–ERB. These feedback loops work in concert to generate a period of 24 h. A highly similar central oscillator exists in *Drosophila melanogaster*, one associate loop stabilizes the primary loop, consisting of CLOCK (CLK)–cycle (CYC) and PER–timeless (TIM; Zhang and Kay, 2010). The core circadian clock genes have been reported to participate in tumorigenesis and metabolic disorders. Early landmark studies have shown that mice carrying mutations of *Per2^m/m^* had reduced apoptotic responses in thymocytes and increased lymphoma occurrence when irradiated (Fu et al., 2002). Similar experiments have been performed in Cry1^−/−^ mice, which exhibits resistance to obesity induced by a high-fat diet (Griebel et al., 2014). Recent studies have shown that BMAL1 can promote tumorigenesis and chemoresistance with MiR-135b (Jiang et al., 2016, 2018). Altogether these studies have supported that circadian clocks are associated with various physiological processes and pathological events. The multiple functions of core circadian oscillator and PCBPs suggests that these two pathways engage in crosstalk with each other.

Our previous screen identified PCBP1, a member of the PCBPs family, as a period-short candidate (Zhang et al., 2009). The CircaDB online database predicts that most PCBP genes are under daily cycling in mouse tissues (Hughes et al., 2009). According to these observations, we hypothesize that PCBP1 plays important role in circadian rhythm and studied the role of PCBPs in circadian machinery.

## Materials and Methods

### Plasmid DNA and Antibodies

Transient expression vectors were generated by inserting cDNA into the multi-cloning site of pcDNA3.1(+)/hygro. Small interfering RNA (siRNA) of PCBP1 and PCBP2 were synthesized as follows: siPCBP1-1, 5'-GCUCCUCUGGUAGGCAGGUUACUAU-3' (forward) and 5'-AUAGUAACCUGCCUACCAGAGGAGC-3' (reverse); siPCBP1-2, 5'-CCGGUGUGACUGAAAGUGGACUAAA-3' (forward) and 5'-UUUAGUCCACUUUCAGUCACACCGG-3' (reverse); siPCBP2, 5'-CCGGUGUGAUUGAAGGUGGAUUAAA-3' (forward) and 5'-UUUAAUCCACCUUCAAUCACACCGG-3' (reverse). The short hairpin RNA (shRNA) expression vectors were obtained by subcloning short DNA sequences into pLKO.1 as follows: shPCBP1, 5'‐ TAAGAGTGGAATGTTAATAAACTCGAGTTTATTAACATTCCACTCTTA-3'; shPCBP2, 5'-CTAGAGGCCTATACCATTCAACTCGAGTTGAATGGTATAGGCCTCTAG-3'. Antibodies used for co-immunoprecipitation included anti-Flag (Cat# F3165, sigma) and anti-HA (Cat# ab9110, Abcam).

### Cell Culture and Transfection

U2OS cells and HEK293T cells were cultured at 37°C in a 5% CO_2_ atmosphere in DMEM supplemented with 10% fetal bovine serum and a mixture of penicillin and streptomycin. Stable shPCBP1 or shPCBP2 cell lines were generated following a previously described protocol (Zhang et al., 2009). Cells were transfected with siRNA and plasmids using RNAi MAX and Lipofectamine 2000 transfection reagent, respectively, according to the manufacturer’s instructions (#13778150, #11668019, Life Technologies).

### Quantitative Real-Time PCR

RNA was extracted from cells and mouse hearts with Trizol reagent according to the manufacturer’s instructions (#15596, Life Technologies). cDNA was generated from RNA using a PrimeScript RT Master Mix Real-time RT-PCR Kit (#RR036A, Takara). Gene expression was analyzed *via* quantitative real-time PCR (qRT-PCR) with a KAPA SYBR FAST qPCR Kit (#KP-KK4601, Kapa Biosystems). The reactions were first incubated at 95°C for 10 min, followed by 40 cycles at 95°C for 10 s, 60°C for 30 s. Gene-specific primer sequences are as follows: hTBP, 5'-GAACCACGGCACTGATTTTC-3' and 5'-CCCCACCATGTTCTGAATCT-3', hPCBP1, 5'-GGACAACACACCATTTCTCCGC-3' and 5'-AGCCTTTCACCTCTGGAGAGCT-3'; hPCBP2, 5'-ATCTGCTGCCAGCATTAGCCTG-3' and 5'-GGTGGTGAACAGCAGAAAGGGA-3'; mTbp, 5'-CAAACCCAGAATTGTTCTCCTT-3' and 5'-ATGTGGTCTTCCTGAATCCCT-3'; mPcbp1, 5'-GGACAACACACCATTTCTCCGC-3' and 5'-AGCCTTTCACCTCTGGAGAGCT-3'; mBmal1, 5'-CTCGACACGCAATAGATGGGA-3' and 5'-CTTCCTTGGTCCACGGGTT-3'; mPer2, 5'-GAAAGCTGTCACCACCATAGAA-3' and 5'-AACTCGCACTTCCTTTTCAGG-3'; mCry1, 5'-GGTTGCCTGTTTCCTGACTCGT-3' and 5'-GACAGCCACATCCAACTTCCAG-3'.

### Luminometry and Luciferase Repression Assays

Human U2OS cells harboring *Per2-dLuc* were grown to confluence in 3.5 cm dishes and were then placed in XM medium and sealed (Zhang et al., 2009). Data were collected in a LumiCycle luminometer at 36°C for 5–7 days; data excluding the first 24-h cycle were analyzed with LumiCycle Analysis software (Actimetrics, Inc.; Liu et al., 2007).

Luciferase repression assays were performed in Corning 96-well white bioassay plates with HEK293T cells as described previously (Liu and Zhang, 2016). The luciferase reporter with tandem E-boxes derived from the Dbp gene was used in the experiments as described previously (Wu et al., 2017).

### Luciferase Complementation Assay

Luciferase complementation assay was used to determine the interaction of proteins as previously described (Liu and Zhang, 2016). The N-terminal and C-terminal luciferase fragment were fused to two different proteins, such as CLOCK and BMAL1, and were then co-expressed as fusion proteins with luciferase fragments in HEK293T cells. Twenty-four hour after transfection, cells were prepared for the Dual-Luciferase Reporter Assay System (Promega).

### Co-immunoprecipitation Assay

HEK293T cells were transfected with Lipofectamine 2000 according to the manufacturer’s instructions (#11668019, Life Technologies). At 24 h after transfection, the cells were washed with PBS buffer and harvested with lysis buffer (50 mM Tris–HCl (pH 7.5), 150 mM NaCl, 1% NP-40) containing protease inhibitor. The lysates were immunoprecipitated with HA or FLAG-agarose beads and the complexes were washed three times with lysis buffer. The immunoprecipitated proteins were separated using 12% SDS-PAGE and transferred to polyvinylidene difluoride (PVDF) membranes (Millipore). Non-specific binding was blocked with 5% skimmed milk in Tris buffer, then the proteins were probed with anti-Flag antibody and anti-HA antibody. Antibody binding was subsequently detected by incubation with secondary antibodies linked to horseradish peroxidase (HRP), and immunoreactive proteins were visualized using an ECL detection kit (Thermo).

### Behavior Measurements

The fly strains Cry16-Gal4 were gifts from Luoying Zhang, and UAS-shMub (THU02931) were from the Tsinghua University Stock Center. All the flies were raised on a standard cornmeal-yeast-sucrose medium and a 12-h light/12-h dark (LD) cycle at 25°C. Male flies (2–3 days after eclosion) were used to monitor locomotor activity levels using the *Drosophila* Activity Monitor system (Trikinetics, Waltham, MA). Individual flies were placed in a glass tube with food, and their activity were recorded. Flies were subjected to an LD cycle with light intensity of 200 lux for 7 days and then 7 days of constant darkness (DD) condition. Data of behavior rhythmicity during the DD condition were analyzed with ClockLab software (Actimetrics, Inc.; Bu et al., 2019).

### Statistical Analysis

In all experiments, unless noted, error bars represent SEM. Statistical analyses were performed using Graph-Pad PRISM (version 7.00, GraphPad Software, Inc.) and JTK_Cycle software (Hughes et al., 2010). Two-sided Student’s *t*-tests were used when only two groups were analyzed. One-way ANOVA with Dunnett’s multiple comparisons test was used when more than two groups were being analyzed, otherwise we used two-way ANOVA with Bonferroni’s multiple comparisons test in some experiments. ^*^p < 0.05, ^**^p < 0.01, and ^***^p < 0.001.

### Ethics Approval Statement

Mice were housed in a specific-pathogen-free (SPF) environment under a 12 h light/dark photoperiod with food/water. All experiments with mice were performed following the guidelines of the Institutional Animal Care and Use Committee (IACUC) at NIBS.

## Results

### PCBP1 Is a Period-Short Modifier for the Mammalian Clock

**Figure 1:**
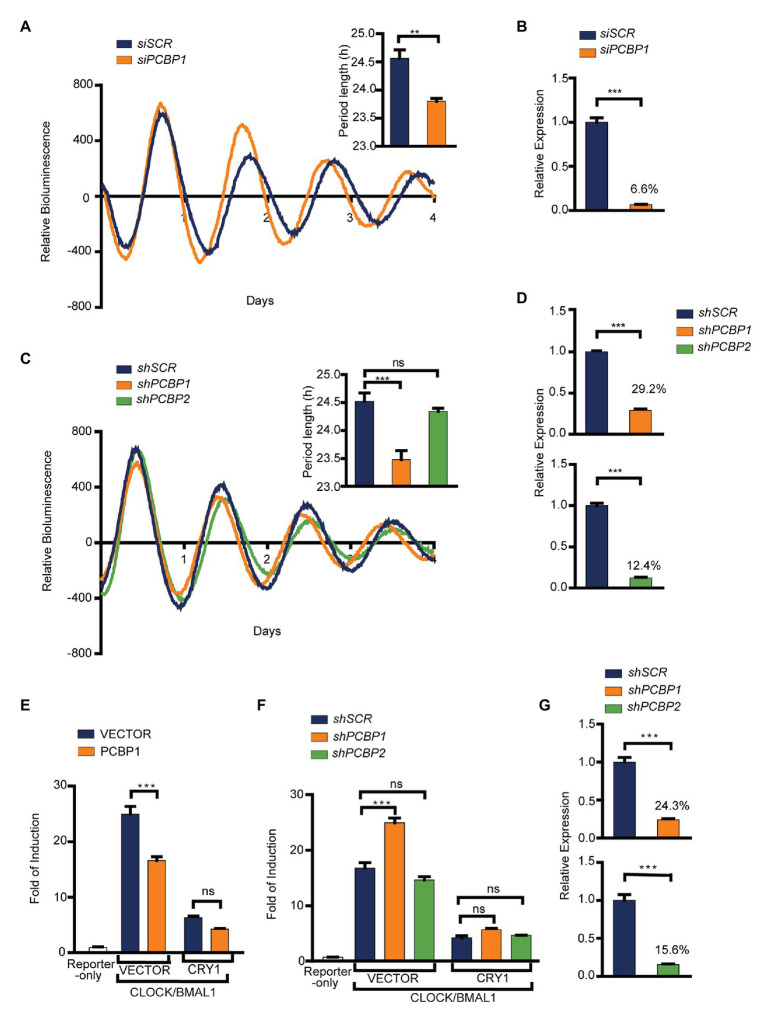
PCBP1 is a period-short modifier for mammalian clocks. **(A)** Knockdown of PCBP1 results in a short period of circadian rhythm in U2OS cells harboring Per2-luc. Top right: Bars show quantitative period change values. **(B)** The knockdown efficiency of siPCBP1. The expression level of PCBP1 was knocked down to 6.6% compared to scramble. **(C)** Knockdown of PCBP1 using short hairpin RNA (shRNA) results in a short period of circadian rhythm in U2OS cells harboring Per2-luc, and the period had no obvious change in shPCBP2 cells. Top right: Bars show quantitative period change values. **(D)** The knockdown efficiency of shPCBP1 and shPCBP2 in U2OS cells harboring Per2-luc. The expression levels of PCBP1 and PCBP2 were 29.2 and 12.4% compared to scramble, respectively. **(E)** A transient transfection luciferase assay was conducted in 293 T cells to show that over-expressed PCBP1 decreased the transcriptional activity of CLOCK/BMAL1. **(F)** A transient transfection luciferase assay was conducted in shPCBP1 and shPCBP2 cells to show the difference in the transcription or repression activity. **(G)** The knockdown efficiency of shPCBP1 and shPCBP2 in 293 T cells. The bioluminescence traces in **(A,C)** were representative of five independent experiments, and the four additional experiments gave similar results. Mean and error bars (SEM) of five independent transfections are shown. Statistical significance was determined using Student’s *t*-tests **(C)**, one-way ANOVA **(A,B,D,G)** or two-way ANOVA followed by Bonferroni’s multiple comparisons test **(E,F)**. (*n* = 5, ^**^p < 0.01, and ^***^p < 0.001).

To investigate whether PCBPs regulate the circadian clock or not, we first knocked down the expression of PCBP1 in U2OS cells containing Per2-dLuc using siRNA. Our results showed that the knockdown efficiency of PCBP1 was 93.4%, and that the clock of PCBP1 knockdown cells had a shorter period (−0.8 h) than scramble control cells (Figures 1A,B), suggesting that PCBPs could modify the mammalian clock. Considering that PCBP1 and PCBP2 share a high level of amino acid sequence similarity (Makeyev and Liebhaber, 2002), we further used a shRNA lentivirus system to knock down the expression of PCBP1 or PCBP2 (Figures 1C,D). The knockdown efficiency of PCBP1 and PCBP2 were 70.8 and 87.6%, respectively (Figure 1D). Interestingly, the circadian period was short in shPCBP1 cells (−1.0 h) like siPCBP1 cells but did not have larger changes in scramble control and shPCBP2 cells (Figure 1C), indicating that PCBP1 but not PCBP2 is a period-short modifier for the mammalian clock in U2OS cells.

To further investigate the roles of PCBP1 and PCBP2 in the circadian clock, we detected the activity of CLOCK/BMAL1 using E-box-based luciferase reporter assay when manipulating the expression of PCBP1 or PCBP2 in HEK293T cells. We looked for associations of transcriptional activity and repression activity with response to PCBP1 by two-way ANOVA including factor interaction. Our results showed that the transcriptional activity of CLOCK/BMAL1 was reduced in PCBP1 overexpressed cells (Figure 1E) but induced in shPCBP1 cells of which the knockdown efficiency was 75.7% (Figures 1F,G). In addition, the transcriptional repression activity of CRY1 was enhanced and weakened in PCBP1 overexpressed cells and knockdown cells, respectively, although with no significance. Two-way ANOVA test showed a significant *Interaction* effect between PCBP1 and CRY1 for the transcriptional activity of CLOCK/BMAL1 (*p* < 0.001). However, the activity of CLOCK/BMAL1 and CRY1 did not have obvious change in shPCBP2 cells (Figure 1F), and the knockdown efficiency of PCBP2 was 84.4% (Figure 1G). Altogether these results showed that PCBP1 was a period-short modifier that appears to be mediated by its impact on the CLOCK/BMAL1 activation.

### PCBP1 Enhances the Interaction of CRY1 With the CLOCK/BMAL1 Complex

**Figure 2:**
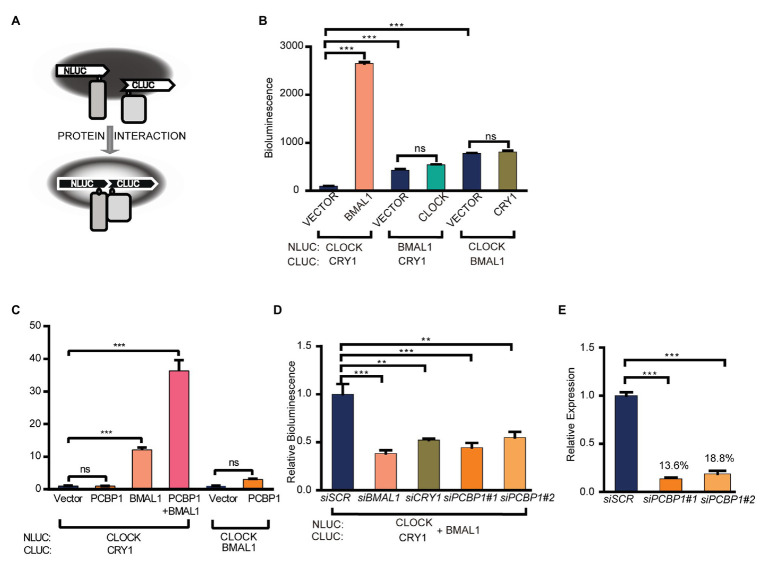
PCBP1 enhances the interaction of CRY1 with the CLOCK/BMAL1 complex. **(A)** schematic diagram of luciferase complementation assay. **(B)** Luciferase complementation assay was used to determine the interaction among CLOCK, BMAL1, and CRY1. **(C)** PCBP1 could not enhance the complex formation of CLOCK-BMAL1 or CLOCK-CRY1, but the formation of CLOCK-BMAL1-CRY1. **(D)** The complex formation of CLOCK-BMAL1-CRY1 in siBMAL1, siCRY1, or siPCBP1 cells. **(E)** The knockdown efficiency of siPCBP1#1 and #2 in 293 T cells. All data are presented as the mean ± SEM from at least three independent experiments. Statistical significance was determined using one-way ANOVA **(C–E)** or two-way ANOVA followed by Bonferroni’s multiple comparisons test **(B)**. (*n* ≥ 3, ^**^p < 0.01, and ^***^p < 0.001).

To investigate how PCBP1 regulates the activity of CLOCK/BMAL1 and CRY1, we conducted a luciferase complementation assay to determine whether PCBP1 affects the interactions between CRY1 and the CLCOK/BMAL1 complex (Figure 2A). CLOCK (or BMAL1) and CRY1 (or BMAL1) were co-expressed as fusion proteins with N‐ and C-terminal luciferase fragments in HEK293T cells, as previously described (Liu and Zhang, 2016). The formation of complexes could produce functional luciferase, the activity of which could be recorded in luciferin-containing medium. Data showed that, compared to the CLOCK/BMAL1 complex and BMAL1/CRY1 complex, the interaction between CLOCK and CRY1 was weakest, although it was enhanced by BMAL1 significantly (Figure 2B). The results indicated that CRY1 could interact with BMAL1 to form the CLOCK-BMAL1-CRY1 complex, which was consistent with a previous report that CRY1 regulated the circadian clock through dynamic interactions with BMAL1 (Xu et al., 2015). Based on this result, we next determined the effect of PCBP1 on the CLOCK-BMAL1 and CLOCK-BMAL1-CRY1 complex. Our results showed that PCBP1 could not enhance the formation of the CLOCK-CRY1 or CLOCK-BMAL1 complex but the formation of the CLOCK-BMAL1-CRY1 complex (Figure 2C), which indicated that the transcriptional activity of CLCOK/BMAL1 was reduced by PCBP1 associated with endogenous CRY1 (Figure 1E).

In addition, we further detected the interaction between CRY1 and the CLOCK-BMAL1 complex in two different siPCBP1 cells in which the expression of PCBP1 was knocked down to 13.6 and 18.8%, respectively, and our results showed that the knockdown of PCBP1 could attenuate the signal of bioluminescence, similar to the knockdown of CRY1 or BMAL1 (Figures 2D,E). All these results suggested that PCBP1 mediated the circadian clock by enhancing the association of CRY1 with the CLOCK/BMAL1 complex, thereby negatively regulating the latter’s activation.

### PCBP1 Interacts With CRY1 *in vitro*

**Figure 3:**
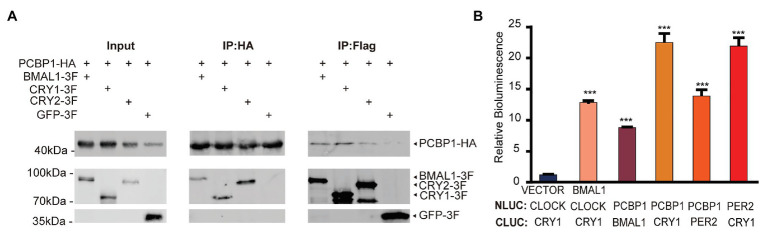
PCBP1 interacts with core clock molecules. **(A)** Co-immunoprecipitation assay shows the interaction between PCBP1 and core clock molecules (CYR1, CRY2, and BMAL1). **(B)** Luciferase complementation assay validates the interaction between PCBP1 and core clock molecules (CRY1, PER2, and BMAL1). All data are presented as the mean ± SEM from three independent experiments. Statistical significance was determined using one-way ANOVA (*n* = 3, ^***^p < 0.001).

We next conducted co-immunoprecipitation and luciferase complementation experiments to study whether PCBP1 could interact with each core clock molecule. The whole cell lysates containing over-expressed PCBP1 with HA-tag and CRY1 (or CRY2, BMAL1) with FLAG-tag were subjected to affinity chromatography on HA or FLAG-agarose beads. The PCBP1 protein complexes were then analyzed by Western Blot (Figure 3A). To avoid non-physiological interaction in over-expressed system in cells, we generated negative control: GFP with FLAG-tag co-expressed with PCBP1-HA. As expected, CRY1 was a major component of the protein complex, although BMAL1 and CRY2 could also copurify with PCBP1. In accordance, the luciferase complementation assay showed that the interaction between PCBP1 and CRY1 was stronger than PCBP1 and PER2 or BMAL1, which was similar with CRY1 and PER2 (Figure 3B). Identification of CRY1, CRY2, and PER2 in the PCBP1 protein complex further confirmed that PCBP1 plays an important role in circadian rhythm modulation.

### PCBP1 Regulating Circadian Rhythm Is Conserved in Evolution

**Figure 4:**
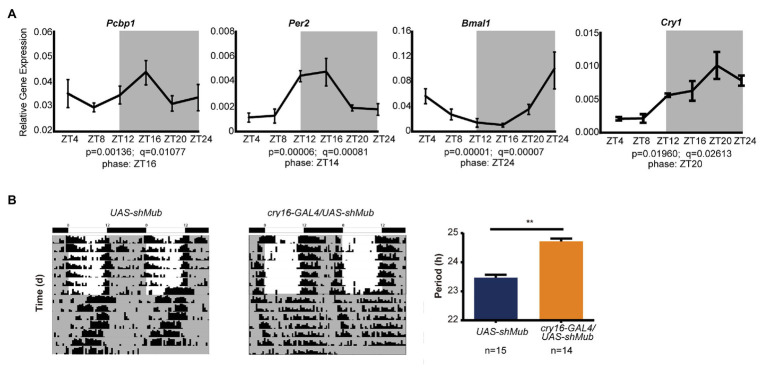
The ability of PCBP1 to regulate circadian rhythm is conserved in evolution. **(A)** The expression of the PCBP1 gene is rhythmic in the mouse heart. The phase of PCBP1 is ZT16, which is similar with PER2 (ZT14) and CRY1 (ZT20), but contrary to BMAL1 (ZT24). All data are presented as the mean ± SEM from at least four independent experiments. The qPCR data were analyzed by JTK_Cycle analysis to determine the rhythmicity and the phases of the peaks (time course threads with both *p* and *q* values less than 0.05, were considered as rhythmic). **(B)** *Drosophila* with conditional knockdown of *mushroom-body expressed* (*mub*), the homolog gene of PCBPs in *Drosophila*, in circadian neurons show a period-lengthening circadian rhythm (middle) compared to wild-type (left). Right: Bars show quantitative period change values Statistical significance was determined using Student’s *t*-tests (*n* ≥ 14, ^**^p < 0.01).

As the CircaDB online database predicts that most PCBP genes are under daily cycling in mouse tissues (Hughes et al., 2009), we determined that the mRNA levels of PCBP1 in mouse hearts exhibited significant circadian oscillation (Figure 4A). The peak mRNA expression level of PCBP1 was at night, which was in accordance with CRY1, PER2 and contrary to BMAL1, which could explain that PCBP1 tended more to interact with CRY1. We further demonstrated that *mub* gene, the homolog of PCBP1 in *Drosophila*, controlled the circadian behavior of fruit flies. We knocked down *mub* in the circadian neurons of *Drosophila via* RNAi interference and recorded the locomotor activity using the *Drosophila* activity monitoring system (Figure 4B). The cry16-GAL4UAS-shMub *Drosophila* showed a significant period lengthening phenotype compared to UAS-shMub *Drosophila*, which suggested that the circadian regulatory mechanism of this post-transcriptional factor is conserved during evolution, although the phenotype was different between mammalian cells and fruit flies.

## Discussion

From the basic TTFL model, both positive and negative elements are important for generating the auto-regulatory feedback loops that drive the circadian rhythm and regulate circadian clock outputs. It has been reported that between 2 and 45% of mouse tissue’s transcriptome display a circadian rhythm (Duffield et al., 2002; Panda et al., 2002; Kornmann et al., 2007; Perelis et al., 2015), and that these clock-controlled genes (CCGs) contribute to multiple physiological and pathological processes including signal transduction, cancer and immune system disorders, and so on (Jagannath et al., 2017). In this study, we found that PCBP1, displaying a circadian expression pattern, was a novel circadian clock regulator, whose function was conserved in evolution.

Poly(rC) binding proteins, known as RNA binding proteins, have multiple functions in biological processes. However, the functions of PCBPs in circadian clock modulation have rarely been reported. Previous genome-wide siRNA screening have showed that PCBPs play important roles in maintaining the period and amplitude in U2OS cells, such as short periods in siPCBP1 or siPCBP3 cells and low amplitudes in siHNRNPK cells (Zhang et al., 2009). In accordance with these findings, our results showed that the circadian period was short in PCBP1 knockdown cells *via* siRNA or shRNA interference and that PCBP2 knockdown cells exhibited no obvious change (Figures 1A,B). Although PCBP1 exhibits the greatest sequence similarity to PCBP2, they may possess non-redundant functions in circadian clock. Immunofluorescence studies revealed that PCBP1 is predominantly in the nucleus (Berry et al., 2006), suggesting PCBP1 works on the circadian clock through transcriptional activation or a repression mechanism. The decreased CLOCK/BMAL1 transcriptional activity and increased CRY1 repression activity by PCBP1 over-expression (Figure 1E) implied that PCBP1 regulates the core circadian transcription-translation negative feedback loop, which validated our hypothesis.

It has been reported that PCBP1 is relevant to tumorigenesis in diverse human cancers (Choi et al., 2009; Shi et al., 2018; Zhang et al., 2020b), and circadian clocks have close interactions with cancer metabolism (Roenneberg and Merrow, 2016; Masri and Sassone-Corsi, 2018). However, the mechanism between these is still unclear. To investigate the underlying mechanism, we further conducted luciferase complementation and co-immunoprecipitation experiments to determine the interaction between PCBP1 and core circadian clock genes. Our results showed that PCBP1 had strong interaction with CRY1 and weak interaction with BMAL1 (Figures 3A,B), because of which PCBP1 could enhance the formation of the CLOCK-BMAL1-CRY1 complex (Figure 2D). In addition, PCBP1 could interact with CRY2 and PER2, which were negative regulators like CRY1 (Figures 3A,B). The mRNA expression level of PCBP1, as expected, showed a circadian pattern consistent with CRY1 and PER2 (Figure 4A), which provide new theoretical basis for chronotherapy (Ju and Zhang, 2020). All these results provided evidence that PCBP1 and circadian clock cross talk with each other, which might be the key to regulate the development of tumorigenesis.

Finally, to define the function of PCBP1 as a novel clock modifier *in vivo*, we recorded the locomotor activity of *Drosophila* with knockdown of *mub* gene, which is the homolog gene of PCBP. Interestingly, we found that *Drosophila* with knockdown of *mub* in circadian neurons showed a circadian rhythm with period-lengthening, the phenotype of which was different from mammalian cells. We considered the difference for two reasons. First, *Drosophila* CRY function is distinct from that of its ortholog in mammals, the major role of which is to repress the transcriptional activity of CLCOK/BMAL1 complex. By contrast, *Drosophila* CRY is a photoreceptor and is responsible for transducing the light input signal to the core repressors PER and TIM (Zhang and Kay, 2010). As such, in *Drosophila*, knockdown of *mub* results in a different phenotype from mammalian cells. Second, in mammalian cells, the PCBP protein family contains five components, PCBP1–4 and HNRNPK, which may interact with each other to affect the circadian period. However, *mub* in *Drosophila* is the only one PCBP homolog gene, *thereby* manipulating circadian rhythm by a different mechanism.

In summary, we identified PCBP1 as a novel circadian clock modifier through interaction with core clock molecules, whose function was conserved in evolution, although the mechanism might be different between mammalian cells and *Drosophila*.

## Data Availability Statement

The original contributions presented in the study are included in the article/supplementary material, further inquiries can be directed to the corresponding author.

## Ethics Statement

The animal study was reviewed and approved by the Institutional Animal Care and Use Committee (IACUC) at NIBS.

## Author Contributions

YW and NL conceived the study. YW, HZ, and NL carried out the experiments and analyzed the data. EZ provided guidance. NL wrote the manuscript. All authors contributed to the article and approved the submitted version.

### Conflict of Interest

The authors declare that the research was conducted in the absence of any commercial or financial relationships that could be construed as a potential conflict of interest.
